# RoCoMAR: Robots' Controllable Mobility Aided Routing and Relay Architecture for Mobile Sensor Networks

**DOI:** 10.3390/s130708695

**Published:** 2013-07-05

**Authors:** Duc Van Le, Hoon Oh, Seokhoon Yoon

**Affiliations:** Department of Electrical and Computer Engineering, University of Ulsan, Ulsan 680-749, Korea; E-Mails: anhduc.mta@gmail.com (D.V.L.); hoonoh@ulsan.ac.kr (H.O.)

**Keywords:** robot relays, mobile *ad hoc* routing, controllable node mobility

## Abstract

In a practical deployment, mobile sensor network (MSN) suffers from a low performance due to high node mobility, time-varying wireless channel properties, and obstacles between communicating nodes. In order to tackle the problem of low network performance and provide a desired end-to-end data transfer quality, in this paper we propose a novel *ad hoc* routing and relaying architecture, namely RoCoMAR (Robots' Controllable Mobility Aided Routing) that uses robotic nodes' controllable mobility. RoCoMAR repeatedly performs *link reinforcement* process with the objective of maximizing the network throughput, in which the link with the lowest quality on the path is identified and replaced with high quality links by placing a robotic node as a relay at an optimal position. The robotic node resigns as a relay if the objective is achieved or no more gain can be obtained with a new relay. Once placed as a relay, the robotic node performs *adaptive link maintenance* by adjusting its position according to the movements of regular nodes. The simulation results show that RoCoMAR outperforms existing *ad hoc* routing protocols for MSN in terms of network throughput and end-to-end delay.

## 1. Introduction

A mobile sensor network (MSN) [1–3] has opened a great promising area for various applications, such as localization, target tracking, disaster recovery, or search and rescue operations in a wild area where communication infrastructures can not be used. In such a network, a stable multi-hop communication is required for the effective operations of mobile sensors.

For example, in search and rescue operations, mobile sensors which are carried by unmanned vehicles or emergency crew members need to send task-related data such as continuous images or real-time videos to the sink (base station) or other mobile sensors to share findings or status of the wounded personnel. Unfortunately, in a practical deployment for those operations, the performance of MSN is significantly degraded due to poor quality wireless links and link breakage, resulting from high node mobility, time-varying channel properties, and geographical constraints (e.g., obstacles). As a result, the network may not be able to provide a desired quality for the end-to-end data transfer.

Due to high node mobility, routing protocols [4] that have been proposed for traditional wireless sensor networks are not suitable for MSNs. Instead, mobile ad-hoc routing protocols (e.g., AODV [5], DSR [6], OLSR [7], and greedy forwarding based schemes [8,9]) may be used for MSNs. However, those protocols try to find a route by taking into consideration only the *given* network topology. As a result, if the selected best path contains a wireless link with a poor quality and an alternative path cannot be discovered in the given topology, the network performance on the path will be limited by the link with the lowest quality. Even in the case that every link on the path initially has a good quality, it is possible that the quality of links can be lowered due to the lengthened distance between communicating peers as nodes move.

In this paper, the use of robotic nodes is considered to overcome the limitations of existing ad hoc routing protocols. More specifically, in order to address of the low performance of MSN and provide a high-quality and mission-critical data transfer service, we propose a novel routing and relaying architecture namely *RoCoMAR* (Robots' Controllable Mobility Aided Ad hoc Routing) that uses controllable mobility of robotic nodes, *i.e.*, RoCoMAR controls the motion of robotic nodes during the network lifetime with the objective of maximizing the network throughput.

To achieve the objective, RoCoMAR repeatedly performs *link reinforcement* process until the required performance is achieved or no more performance gain can be obtained. In the link reinforcement process, the link with the lowest quality on the path is identified and a robotic node is placed at an optimal position to create high quality relay links that will replace the poor quality link. In this paper, PDR (packet delivery ratio) is used as an indicator of a link quality. On duty as a relay, the robotic node also performs *adaptive link maintenance*, where the robotic node keeps adjusting its position according to the network topology change due to movements of regular MSN nodes (e.g., sensor devices that are carried by unmanned vehicles or personnel such as crew members). The robotic node resigns as a relay if the quality of the original link, which used to have a poor quality, becomes high enough due to node mobility. In other words, a robotic node adaptively determines not only its position but also whether or not it should stay as a relay according to the network topology change.

**Figure 1.**
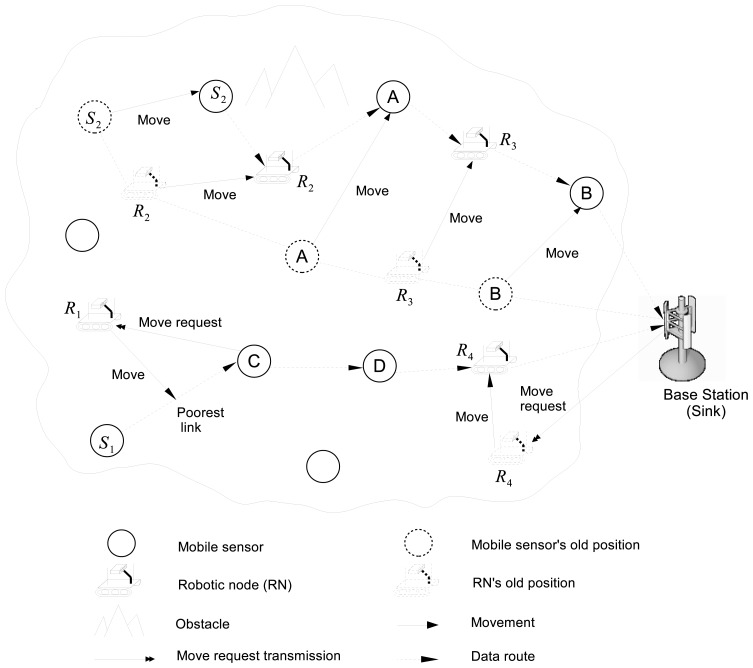
An example of mobile sensor network with robotic relays.

Figure 1 illustrates the overview of RoCoMAR. As shown in the example in Figure 1, the link with the lowest quality is identified and replaced with new links. For example, on the path from node *S*_1_ to the base-station (sink or command center), the link between node *S*_1_ and node *C* has the poorest quality. Then, a robotic node *R*_1_ is relocated between node *S*_1_ and node C and two new relay links will be created via node *R*_1_. Also note that there is another robotic node, *R*_4_, which is already working as a relay on the path. If the team of MSN nodes moves away from the base station, more robotic nodes will be added on the path to keep the team connected to the base-station.

As also shown in Figure 1, each robotic node keeps adjusting its position according to the movements of its neighboring nodes. For example, robotic node *R*_3_ autonomously relocates itself to an optimal position for relaying as node *A* and *B* move. A simple yet effective heuristics for determining the new position of a robotic node is to place it in the mid-point of the poor link, since it will result in maximization of the link quality and minimization of the transmission power [10]. Robotic node can be placed in another position if it cannot be placed at the midpoint of the link due to geographical constraints. For instance, robotic node *R*_2_ is placed at another position due to an obstacle. The robotic node may resign as a relay, if a relay is no longer necessary. For example, if the distance between node *A* and *B* becomes close after movements and a high quality wireless link can be established between them, robotic node *R*_3_ will resign as a relay.

It is worthwhile to note that there have been studies that considered controllable mobility of nodes in mobile wireless sensor networks due to its potentials in many applications. For example, nodes' controllable mobility can be used to reduce energy consumption while relaying data packets in a multi-hop fashion [11], to monitor electro-magnetic fields [12], and to deploy sensors at optimal positions for improving network coverage [13,14]. There also have been studies that use controllable mobility to design routing protocols [11,15,16]. However, those works focus on minimizing the total energy consumption of nodes for communication and movements rather than maximizing the network throughput. Furthermore, those works assume that all nodes in the network are robotic agents, which make them less applicable to a practical mobile sensor network application considered in this paper (e.g., emergency search/rescue and military reconnaissance/surveillance operations).

The rest of the paper is organized as follows. In Section 2, we present the related work and contrast with proposed algorithms. The system model and general assumptions are presented in Section 3. Section 4 presents the detail algorithms of RoCoMAR and an analytical model. In Section 5, simulation setup and analysis are presented. Finally, Section 6 concludes the paper.

## 2. Related Work

In this section, we present an overview of recent studies related to mobile sensor networks and controllable mobility based routing protocols.

### 2.1. Mobile Sensor Networks

Recently, many studies have considered applications of MSN where mobile sensors are equipped with sensing, computing and communication capabilities, and powerful batteries for complex processing tasks. For example, a network of distributed mobile sensors can be developed as a solution for searching immobile human victims, following gradients of temperature, investigating dangerous area [1–3].

One distinct advantage of mobile nodes comes with their possibly controlled mobility, which has been extensively studied to address research challenges in MSNs [13,14,17–19]. For example, in [13,14], controllable mobility was used to enhance the coverage in distributed wireless sensor networks. Initially, MSN nodes are placed at random positions which do not always lead to the effective coverage. Then, controllable mobility is used to move sensors to optimal positions such that the maximum coverage is achieved. The authors in [13,18] studied the use of mobile robots for target exploration and localization.

In [19], controllable mobility of mobile robots, which can pick, carry and unload sensor nodes has been used to assist sensor replacement in a large scale static sensor network. When failed sensors are detected, the mobile robots first move to the positions of the failed nodes, then unload and replace failed sensors.

Mobile robot networks where all nodes are robotic nodes have also been studied for decomposition, allocation, search&rescue, and planetary exploration which are traditionally performed by human teams [20,21]. The search and rescue operations can be executed using a system of cooperative multiple mobile robots which are deployed in teams or groups [20]. The multiple mobile robots perform distributed sensing, monitoring or surveillance in a large area, and pass the collected data to a command center for further analysis through cooperative communication. In [21], a distributed cooperative communication algorithm was proposed for *ad hoc* networked mobile robots.

Our work differs from those works in that the objective of RoCoMAR is to improve the performance of routing protocols for MSN using controllable mobility of robotic nodes rather than coverage maximization, exploration and localization.

### 2.2. Controllable Mobility Aided Routing Protocols

Some traditional routing protocols proposed for mobile ad hoc networks (e.g., AODV [5], DSR [6], OLSR [7], LAR [22]) and energy-conserving protocols [23,24] can be applied in MSNs [25]. However, those protocols do not consider controllable mobility as a network parameter when they determine the routes and forward data packets. In contrast, in this paper, our focus is on designing an ad hoc routing architecture that exploits the nodes' controllable mobility.

There have been studies that considered controllable mobility in designing routing protocols for MSNs [10,11,15,16]. These protocols first adopt existing multi-hop routing protocols to discover an initial path from a source node to a destination node. Then, nodes on the path except for the source node and the destination node are moved to the positions such that some specific objectives are achieved. For example, the study in [10] showed that the optimal configuration of node positions for energy efficiency can be achieved when all relay nodes are evenly spaced along a line connecting source and destination nodes.

In [11], the optimum number of hops for minimizing total transmission power of the route from the source node and the destination node were investigated, and then a routing algorithm called optimal hop count routing (OHCR) was proposed, which is based on the calculated optimum number of hops. The authors further proposed another routing algorithm called MPoPR that minimizes transmission power over progress. After a route between source and destination nodes is discovered, the relay nodes on the route can be moved to their computed optimal positions directly or in rounds to minimize energy consumption.

A controllable mobility based geographic routing, CoMNet, was proposed in [15]. CoMNet takes advantages of node mobility to improve routing energy consumption while ensuring network connectivity. Both of transmission cost and mobility cost are optimized in route establishment process. In [16], the nodes's position information was incorporated in the route discovery mechanism of AODV to build the path between source and destination nodes which minimizes the total traveling distance of relay nodes. After the best route is found, the relay nodes of route move to positions which are evenly spaced the straight line connecting source and destination nodes.

However, those works differ from ours in that they assume that all nodes in the network are robotic agents, *i.e.*, all movements of nodes are controllable. This assumption not only requires a high system cost, but also is not appropriate for applications considered in this paper where considerable number of sensor nodes are carried by unmanned vehicles or humans and cannot be controlled for routing purpose (e.g., search&rescue crew members and a group of unmanned vehicles for surveillance). Moreover, those works focus on minimizing energy consumption for communications. In contrast, maximizing throughput is the major objective of our work, which is important for network applications under consideration (e.g., high-rate sensing and multimedia communications).

## 3. System Model

RoCoMAR is designed for MSNs where mobile sensors are deployed to monitor and track the area of interest for various applications such as emergency search & rescue and environment monitoring operations. The MSN under consideration consists of multiple regular mobile sensors (hereafter referred to as regular nodes or simply nodes) and autonomous robotic nodes (or RNs) for relaying communication data.

Regular nodes are sensors carried by unmanned vehicles or humans (e.g., emergency crew members). Note that the mobility of regular nodes depends on the operation they perform. For example, regular nodes may cooperate with other regular nodes in a given area to track a target, or perform a search&rescue, which leads to the group mobility. On the other hand, the self-propelled robotic node controls its movement based on network status in order to improve the network performance. Once a robotic node decides to move to a target position, it uses an equipped navigation system to travel.

For simplicity, in this paper, it is assumed that a robotic node moves towards the target position on a straight line with a given speed. Obstacle avoidance while moving is not a targeted problem of this work, and for this problem existing navigation algorithms [26–30] can be considered.

It is also assumed that mobile sensors are equipped with sensing devices and a video camera to collect information from the monitored area. Collected data include video frames that are encoded and streamed to the sink for further analysis along with other user data. For example, video frames can be encoded using MPEG-4 technology that can control the output data rate from 9.6 Kbps to 1.024 Mbps [31].

Mobile sensors and robotic nodes are both equipped with a high bandwidth communication device. They can use IEEE 802.11b/a/g based communication devices which can provide a data rate of 1–54 Mbps (can be higher than 100 Mbps with IEEE 802.11n).

It is also assumed that each node can know its geographic position using positioning systems, such as global positioning system (GPS) or other localization technologies [32,33]. Note that RoCoMAR can work only using the relative positions of nodes. For example, if a RN is equipped with devices (e.g., laser radars and beam lasers) that can measure the distance and direction to other nodes, it can readily approach the midpoint position between two nodes.

## 4. RoCoMAR Algorithm

In this section, we describe the algorithms of RoCoMAR in detail. The RoCoMAR is comprised of *link reinforcement algorithm* and *adaptive link maintenance* algorithm, which allow RNs (robotic nodes) to be placed as relays at the appropriate positions and to adjust their positions and movements according to the change of network topology. Note that those algorithms work independently with route discovery algorithm. Therefore, RoCoMAR can use any existing routing protocols (e.g., AODV, DSR, and OLSR) to discover and establish the initial route from the source and destination.

We first describe the link reinforcement algorithm, and then extend the discussion to the adaptive link maintenance algorithm. We also describe the PDR (packet delivery ratio) based link quality estimation, which is used in link reinforcement and adaptive link maintenance algorithms to quantify the quality of wireless links. Finally, we numerically analyze the link quality gain obtained by the link reinforcement processes.

### 4.1. Wireless Link Reinforcement

In RoCoMAR, each node maintains two lists of geographical positions: one list for its neighboring nodes, and another for its one-hop and two-hop neighboring RNs. In order for nodes to obtain the two-hop neighboring RNs' information, each node in the network appends the one-hop RN information to its *Hello* message, which also contains the position of itself and is periodically broadcasted.

Suppose that the source node *S* needs to send data packets to the destination node *D*. After node *S* finds a route using AODV (or using other ad hoc routing protocols), node *S* sends data packet to node *D* through this route. When a node receives a data packet from the predecessor on the path, it appends the current PDR value of the link with its predecessor to the header of the data packet (PDR value is used to estimate the link quality, and the calculation of PDR will be discussed in detail in Section 4.3). When node *D* receives a data packet, it extracts the PDR values of all links on the path and calculates the end-to-end PDR value of route, *R_ete_*,which is

(1) $$R_{\text{ete}}=\prod_{i=1}^{n}R_{i}$$

where *n* and *R_i_* represent the number of links on the path and the PDR value of *i^th^* link. Note that *R_ete_* is a simplified approximation of the end-to-end PDR assuming subsequent transmissions are independent.

Then, node *D* compares the current value of *R_ete_* with a required PDR value, *R_req_*, which is specified by the user application. If the current end-to-end PDR value of the route is less than *R_req_*, the node *D* determines the link that has the lowest PDR value (or the poorest link). Then, node *D* sends a reinforcement request *(REI-REQ)* message to the data-receiving node of the poorest link, which is referred to as REI (reinforcement) node hereafter. Upon the reception of *REI-REQ* message, the REI node sends an *ACK* message to confirm the reception. If node *D* does not receive the *ACK* after *k* time retransmissions of REI-REQ message, it selects another link to reinforce. If the node *D* receives the *ACK* message to the REI-REQ message, it waits for reinforcement reply *(REI-REP)* message, which indicates whether or not the placement of new robotic relay has been successfully performed.

Upon receiving *REI-REQ*, the REI node determines an optimal position where a new RN should be relocated. RoCoMAR uses a simple yet effective heuristics that places a RN in the midpoint of the poor link, which results in maximization of the link quality and minimization of the transmission power [10]. Robotic node can be placed in another position if it cannot be placed at the initially determined position due to obstacles or other geographic features. The algorithm for placing a robotic relay avoiding obstacle is out of the scope of this paper, and in this paper it is assumed that the RN can be relocated to the midpoint.

After the REI node determines the position of new relay, it looks up the list of neighboring RNs and chooses a RN that is closest to the position where a new relay should be placed. When a RN is selected, the REI node sends a move request *(MREQ)* message which contains the network address of its predecessor on the data path and the position to which the RN should move. If the REI node does not receive *ACK* to *MREQ* after *h* time retransmissions, it selects another RN in the list unless the list is empty. If the REI node could not select any RN, it informs node *D* of this failure using *REI-REP* message.

When a RN receives *MREQ*, it starts moving immediately to the position that is indicated in *MREQ*. Upon arrival at the dictated position, the RN sends an *CHG-NEXT-HOP* (change next hop) message to the predecessor node of the REI node on the path, so that the predecessor node updates its routing table and forwards the data packets to the RN from that time on. The RN also adds a route entry that contains the REI nodes as the next hop. When the REI node receives the data packet from RN, it sends a REI-REP message to node *D* indicating that a new relay node is successfully added.

Each RN has two states: *Stand-by* and *Relaying*. A RN is initially in *Stand-by* state, and switches to *Relaying* state after it sends a *CHG-NEXT-HOP* message to the predecessor node. If a RN receives multiple *MREQs*, it accepts the first request and discards others. However, in order to allow a RN to relay data for multiple flows that share the same link, a RN accepts a relay request for the same link for which it is already working as a relay. When the data transmission on path finishes or it receives a *RES-REQ* (Resign Request) from the REI node, the RN switches to *Stand-by* state.

**Figure 2.**
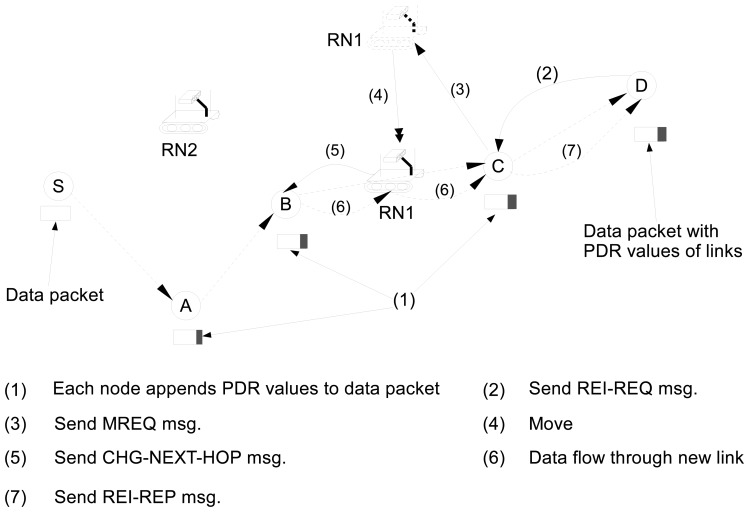
An example of link reinforcement process.

Figure 2 illustrates an example of the reinforcement process. As shown in the example of Figure 2, each node on the path (e.g., *A*, *B* and *C*) appends the PDR value of its link to the data packet header (1). When node *D* receives the data packet which consists of the PDR values of links, it calculates *R_ete_*. Assume that *R_ete_* is less than *R_req_*, and the link between *B* and *C* is the link with the lowest quality on the path. Then node *D* sends a *REI-REQ* to node *C* to request for a new relay node (2). When node *C* receives *REI-REQ* (2), it becomes a REI node and selects *RN*1 as a new relay and sends *MREQ* to RN1 (3). Upon receiving *MREQ*, the RN1 moves to the midpoint position of the link between node *B* and node *C* (4). When the RN1 arrives at position of the midpoint of link between node *B* and node *C*, it sends the *CHG-NEXT-HOP* message to node *B* (5) and adds itself to the route from node *S* to node *D*. Node *B* updates its routing table and starts forwarding the data packets to RN1 (6). When node C receives the first data packet from RN1 (6), it sends a *REI-REP* that indicates that a new relay node is successfully added (7).

The repeated link reinforcement process algorithm for a destination node is presented in Algorithm 1. Let *R_c_* and *R_b_* denote the current end-to-end PDR value and the end-to-end PDR value before a RN is added to the route, respectively. *G* denotes the set of nodes in the network. The *link reinforcement* process is repeatedly performed until the required end-to-end PDR, *R_req_*, is achieved or no more gain on PDR can be obtained. As presented in Algorithm 1, the destination node *D* uses *R_c_*, *R_b_*, and *R_req_* value to determine whether or not it initiates a new link reinforcement process.

In order to initiate a new reinforcement process, *R_c_* should be less than *R_req_*, and the PDR gain by the latest reinforcement should not be negligible, *i.e.*, *R_c_* ≥ *R_b_* (1+*ξ*) as shown in line 5 in Algorithm 1, where *ξ* represents a system parameter for the PDR gain ratio. The value of *ξ* affects the network performance. A too small value of *ξ* can result in a lot of unnecessary reinforcement processes without a significant performance gain. On the other hand, a large value of *ξ* may prevent the network from placing a new relay node even when some degree of performance gain is expected. If *R_c_* is less than *R_b_* (*i.e.*, placing a new relay actually lowers the end-to-end PDR), then the newly added RN is removed from the route. Note that node *D* iteratively checks whether or not it can initiate a new reinforcement process every time interval *t_rei_*.


**Algorithm 1** Repeated Link Reinforcement Process-Run at destination node.
1:*R_c_*← Ø;2:*R_b_*← Ø;3:**while** connection is ACTIVE do4: 
$R_{c}=\prod_{i=1}^{n}R_{i}$▹ *R_i_*: PDR value of *i^th^* link5: **if**
*R_c_* < *R_req_* && *R_c_* ≥ *R_b_*(1+*ξ*) **then**6:  *R_b_* =*R_c_*;7:   (*u*,*v*) = Determine poorest link;▹ *u*,*v* ∈ *G*8:  Initiate a link reinforcement process (*v*);9: **else if**
*R_c_* ≥ *R_req_***then**10:  *R_b_*← ø;11:  Pause for time(*t_rei_*);12: **else if**
*R_c_* ≤ *R_b_***then**13:  *R_b_*← ø;14:  Remove newly added robotic relay node;15:  Pause for time(*t_rei_*);16: **end if**17:**end while**


### 4.2. Adaptive Link Maintenance

RoCoMAR enables the network not only to keep high quality links but also to prevent links from being broken, which results in a significant improvement of routing protocol performance. In this subsection, we describe RoCoMAR's adaptive link maintenance algorithm, which allows RNs to adjust their positions according to the network topology change and to prevent link breakage by working with the link reinforcement algorithm.

RNs perform the adaptive link maintenance when they work as relay nodes. After each RN becomes a relay node, it periodically updates the positions of its predecessor (referred to as PRE node hereafter) and its next-hop node (referred to as NEXT node hereafter) on the path from the source to the destination, using the information obtained from the received *Hello* messages. Then, the RN relocates itself to the midpoint position of the segment between its PRE and NEXT nodes at every *t_alm_* seconds. The system parameter *t_alm_* is set considering the speed of the RN's PRE and NEXT nodes and the speed of RN itself such that the links of RN with the PRE and NEXT nodes would not be broken or the links' PDR values would not become less than a specific threshold value.

It is possible that the PRE and NEXT nodes of a RN become close each other as they move, and hence the PRE and NEXT nodes could establish a high quality direct link. RoCoMAR's adaptive link maintenance allows a RN to detect this situation and resign as a relay. More specifically, the NEXT node calculates the PDR value of the potential link with the PRE node based on *Hello* messages received from the PRE node. This PDR value is added to the NEXT node's *Hello* messages so that the RN can also know the PDR value. When the PRE and NEXT nodes can have a direct link with a consistently high quality (*i.e.*, placing a relay node is unnecessary or even wasteful), the RN sends a *CHG-NEXT-HOP* message to its PRE node which leads to establishment of a direct link between the PRE and NEXT nodes.

Note that the PRE and NEXT nodes may be either RNs or regular nodes. In other words, in RoCoMAR, multiple RNs can work as relay nodes in a row between two regular nodes. This feature of RoCoMAR is particulary useful when one or a group of nodes moves far away (e.g., for searching or following some events in the operation) and still needs a communication link with the rest nodes in the network. Also note that when those nodes are returning to the rest nodes, the RNs detect this and *adaptively* resign as a relay.

**Figure 3.**
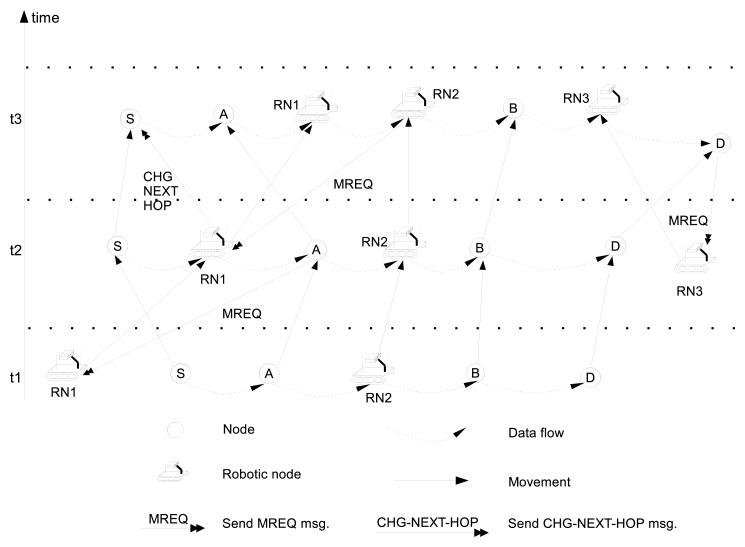
An example of an adaptive link maintenance process over time.

Figure 3 illustrates an example of the adaptive link maintenance process for the route from node *S* to node *D* as the network topology changes over time. As shown in Figure 3, at time *t*_1_, RN2 is a relay node between node *A* (PRE node) and node *B* (NEXT node). RN2 periodically updates its position to follow the midpoint position of node *A* and *B*. In addition, as shown in Figure 3, at time *t*_2_ RN1 becomes a relay node of the link between node *S* and node *A* after it receives a *MREQ* from node *A*. After some time, RN1 detects that PDR value of the link between node *S* and *A* is greater than the required PDR value, and RN1 resigns as a relay and switches its state to *Stand-by*. At time *t*_3_, as node *A* and node *B* move apart, the quality of link between node *A* and RN2 becomes poor, and RN2 sends a *MREQ* message to RN1 to request to become a relay. Then, RN1 becomes a new relay between node *A* and RN2 and moves to the midpoint of them and RN2 also moves accordingly to the midpoint of RN1 and node *B*.

The link reinforcement and adaptive link maintenance algorithms for a RN is presented in Algorithm 2. *R*(*u*,*v*) denotes the PDR value of link between *u* (data sender) and *v* (data receiver). Also, mid (*u*,*v*) is defined as the midpoint position of node *u* and *v*. As shown in Algorithm 2, when the *R*(*u*,*v*) is equal to or greater than *R_req_*, which is the end-to-end PDR value required by the user application, the RN resigns by sending the *CHG-NEXT-HOP* message.


**Algorithm 2** Adaptive Link Maintenance and Reinforcement Algorithm-Run at RN
1:*R*(*u*,*v*) ← ø;▹ *u*,*v* ∈ *G*2:**while**
*true*
**do**3: **while**
*state* == *Stand-by*
**do**4:  **if** Receive a *MREQ* from *v*
**then**5:   Obtain position information of *u* and *v*;6:   Move to *mid*(*u*,*v*);7:   Send a *CHG-NEXT-HOP* to *u*;8:   *State* = *Relaying*;9:  **end if**10: **end while**11: **while**
*State* == *Relaying***do**12:  Pause for time(*t_alm_*);13:  Obtain *R*(*u*,*v*) from *v*;14:  **if**
*R*(*u*,*v*) < *R_req_*
**then**15:   Move to *mid*(*u*,*v*);16:  **else**17:   Stop at present position;18:   Send a *CHG-NEXT-HOP* to *u*;19:   *State* = *Stand-by*;20:  **end if**21: **end while**22:**end while**


In summary, for improving the quality of data transfer in the route, RoCoMAR first performs the link reinforcement process to identify the poorest link and to place one RN in the midpoint position of the link. After the RN is successfully placed in the midpoint position, it works as a relay node on the route by continuously performing the adaptive link maintenance in order to adjust its position and movement according to the network topology change.

### 4.3. PDR-Based Link Quality Estimator

In RoCoMAR, each node estimates the quality of links that it shares with its neighboring nodes. The PDR based link quality estimation method [34], which is widely used due to its simplicity and accuracy, is used for this paper to estimate the quality of a link.

More specifically, if the sender sends *s* probe packets during a time window, *w*, and the receiver receives *r* probe packets, the PDR of link becomes *PDR*(*w*) = *r*/*s*. Each node updates PDR values of its links with its neighboring nodes at every *w*. In RoCoMAR, *Hello* messages are used as probe packets to calculate PDR values of links.

Note that the size of time window, *w*, affects the accuracy of an estimated PDR value. With a larger value of *w*, a more accurate PDR value can be obtained. However, if *w* is too large, the RoCoMAR may not promptly react to the network topology change.

On the other hand, with a small value of *w*, RoCoMAR can handle the change of a link quality more quickly. However, in this case, a short-period fluctuation of PDR value may result in unnecessary link reinforcement process, which will cause a network overhead and eventually lower the network performance.

Therefore, in order to consider both the accuracy of PDR value and the promptness in reaction, we use a window based approach that also uses previous PDR values, that is

(2) $$R_{i}=\alpha *R_{i}^{w}+\left(1-\alpha \right)*R_{i-1}$$

where *R_i_* is the estimated PDR value of the link at the current time period, $R_{i}^{w}$ is the sampled PDR value which is obtained at the current window (*i.e.*, at the *i^th^* window), *R_i_*_−1_ is the PDR value which was estimated at (*i* − 1)*^th^* time period, *α* is a system parameter and 0 < *α* < 1.

### 4.4. Link Quality Gain by using Robotic Relays

One of major advantages of applying RoCoMAR to MSN is that RoCoMAR can greatly improve the quality of links in a mobile environment by using multiple robotic nodes. In order to gain analytical insight into the link quality gain of RoCoMAR, we consider a simple numerical model. To facilitate discussion and simplify the problem, we assume that the network is unsaturated, and hence the packet loss due to inter-node interference is negligible or can be recovered by using ARQ (Automatic Repeated reQuest) or other MAC technologies without significantly impairing the network throughput and packet delivery ratio.

As shown in [35], the received signal strength is dominated by the distance from the transmitter to the receiver. More specifically, as the distance between the transmitter and receiver becomes longer, the minimum energy required for a successful data packet transmission sharply increases. The average reception power, *P_r_*, at a receiver from the transmitter with the distance *d* in free space is expressed by the Friis equation as $P_{r}=\frac{P_{t}G_{t}G_{r}\lambda^{2}}{{\left(4\pi d\right)}^{2}}$, where *P_t_* is transmitted power, *G_t_* and *G_r_* are the transmitter and receiver antenna gain respectively, both of which are assumed to be 1 for simplicity. *λ* is the wavelength of signal carrier in meters.

Then, the received signal to noise ratio, *SNR*, in an AWGN (Additive White Gaussian Noise) channel, becomes

(3) $$\text{SNR}=\frac{P_{r}}{P_{n}}=\frac{P_{t}\lambda^{2}}{P_{n}{\left(4\pi d\right)}^{2}}$$

where *P_n_* = *WKT* is thermal noise power [36], in which *K* = 1.38×10^−23^*J*/*K* is Boltzmann constant, *T* is room temperature in Kelvins (typically standard temperature of 290*K*) and *W* is signal bandwidth in Hertz.

Assuming that DBPSK (Differential Binary Phase Shift Keying) is used as the modulation scheme, as discussed in [37], the bit error probability, *P_be_*, for the AWGN channel is given by

(4) $$P_{be}=\frac{1}{2}e^{-\frac{E_{b}}{N_{0}}}$$

where *E_b_* is the energy per bit and *N*_0_ is the noise power spectral density. Depending on modulation scheme, $E_{b}/N_{0}=\frac{\text{SNR}}{M}$, where *M* is the number of information bits per symbol. For DBPSK modulation scheme, *M* = 1. Then, from Equations (3) and (4), bit error probability, *P_be_*, becomes

(5) $$P_{be}=\frac{1}{2}e^{-\text{SNR}}=\frac{1}{2}e^{-\frac{P_{t}\lambda^{2}}{\text{WKT}{\left(4\pi d\right)}^{2}}}=\frac{1}{2}e^{-\frac{\Gamma }{d^{2}}}$$

where $\Gamma =\frac{P_{t}\lambda^{2}}{\text{WKT}{\left(4\pi \right)}^{2}}$.

Let *m* denote the length of a data packet in bits. Also, assume that bit errors in a packet follows an uniform distribution with the probability of *P_be_* for simplicity. Then, from Equation (5), the probability of a successful packet transmission, *P_ps_*, can be obtained as

(6) $$P_{ps}={\left(1-P_{e}\right)}^{m}={\left(1-\frac{1}{2}e^{-\frac{\Gamma }{d^{2}}}\right)}^{m}$$

Also, let *R_u,v_* denote the packet delivery ratio (PDR) of the link between arbitrary node *u* and *v*. Then, for long-term packet transmissions, *R_u,v_* can be approximated by the probability of a successful packet transmission, *P_ps_*. That is

(7) $$R_{u,v}\approx {\left(1-\frac{1}{2}e^{-\frac{\Gamma }{d_{\left(u,v\right)}^{2}}}\right)}^{m}$$

where *d*_(*u*,*v*)_ represents the distance between node *u* and node *v*.

Now, consider a route that has *n* links *i.e.*, there are *n*+1 nodes on the route. Assume that the *i^th^* link is formed by node *i* and node (*i*+1) on the route, where 1 ≤ *i* ≤ *n*. Then, the end-to-end PDR of the route is

(8) $$R_{\text{ete}}=\prod_{i=1}^{n}R_{i,i+1}$$

where 1 ≤ *i* ≤ *n*.

Suppose that *j^th^* link on the route has the lowest quality and this link is reinforced by *l* robotic nodes (where 1 ≤ *j* ≤ *n*). Then, the original link quality of the *j^th^* link is $P_{j,j+1}^{\text{ori}}={\left(1-\frac{1}{2}e^{-\frac{\Gamma }{d_{\left(j,j+1\right)}^{2}}}\right)}^{m}$. Also, after *l* reinforcement processes, the quality of the *j^th^* link becomes

(9) $$R_{j,j+1}^{\text{rei}}={\left(1-\frac{1}{2}e^{-\frac{\Gamma {\left(l+1\right)}^{2}}{d_{\left(j,j+1\right)}^{2}}}\right)}^{m\left(l+1\right)}$$

From Equations (8) and (9), the end-to-end link quality gain, *G_q_*, for the route due to the adding number of relay nodes of *l*, can be obtained. That is

(10) $$G_{q}=\frac{\left(\prod_{\underset{i\neq j}{i=1}}^{n}R_{i,i+1}\right)R_{j,j+1}^{\text{rei}}}{\prod_{i=1}^{n}R_{i,i+1}}=\frac{\left(\prod_{\underset{i\neq j}{i=1}}^{n}R_{i,i+1}\right)R_{j,j+1}^{\text{rei}}}{\left(\prod_{\begin{array}{l} i=1\\ i\neq j \end{array}}^{n}R_{i,i+1}\right)R_{j,j+1}^{\text{ori}}}=\frac{R_{j,j+1}^{\text{rei}}}{R_{j,j+1}^{\text{ori}}}\approx \frac{\left(1-\frac{1}{2}e^{-\frac{\Gamma {\left(l+1\right)}^{2}}{d_{\left(j,j+1\right)}^{2}m\left(l+1\right)}}\right)}{\left(1-\frac{1}{2}e^{-\frac{\Gamma }{d_{\left(j,j+1\right)}^{2}m}}\right)}$$

**Figure 4.**
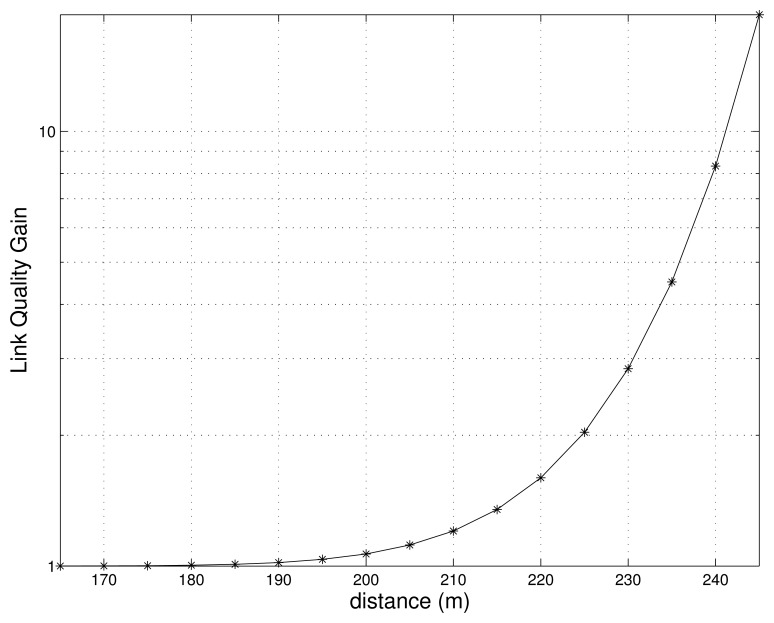
Link quality gain.

The Figure 4 shows the relationship between the quality gain obtained and the distance from the transmitter to the receiver. It is assumed that one relay node is added to the route, *i.e.*, *l* = 1. In addition, we use the transmitted power of *P_t_* = 1*mW*, carrier frequency of *f* = 2.4*GHz*, signal bandwidth of *W* = 54*MHz*, and packet length of *m* = 12,000*bits*, (*i.e.*, 1500*Bytes*) for our evaluations. As shown in Figure 4, the quality gain tends to increase as the distance increases. However, quality gain is 1 when the distance is less than 200 m, which implies that no extra relay nodes are necessary.

## 5. Performance Study

In this section, we first present a simulation architecture namely ConMoSim that can simulate nodes' controllable mobility in a network environment. Then, we evaluate the performance of RoCoMAR by comparing it with other on-demand, proactive, and geographical forwarding based ad hoc routing protocols, such as AODV, DSR, OLSR, and Greedy Forwarding Routing (GFR).

### 5.1. Controllable Mobility Simulator

It is obvious that algorithms using controllable mobility need to be simulated and tested for various scenarios before they are actually applied for real world situations. Unfortunately, existing network simulators do not support controllable mobility. In order to evaluate the performance of RoCoMAR, we developed ConMoSim, a controllable mobility simulation architecture that can operate with a network simulator. ConMoSim is designed to co-operate with a well-known network simulator, QualNet.

**Figure 5.**
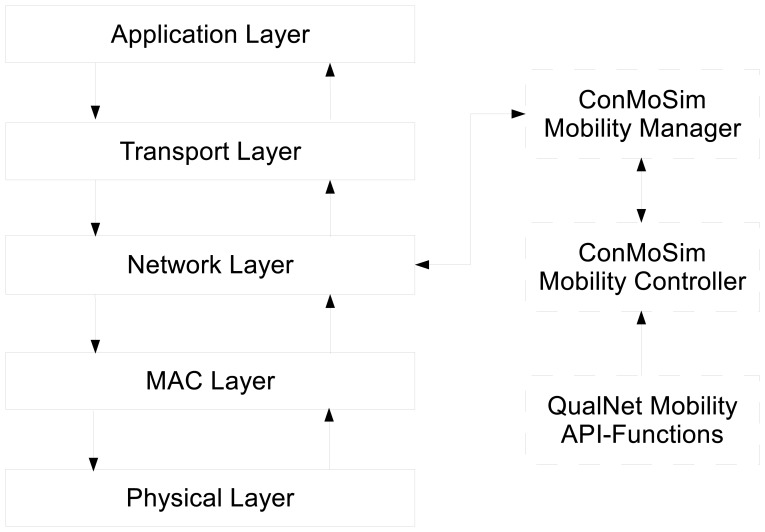
The structure of ConMoSim.

Figure 5 shows the structure of ConMoSim and how the information and data are exchanged. As shown in Figure 5, ConMoSim consists of a mobility manager, an event based mobility controller and interfaces to communicate with other modules of QualNet.

The mobility manager determines whether or not the node moves and, if it decides to move, the speed and direction based on given objectives of the network and the collected information from the network protocol stack *i.e.*, the current network situation. In our protocol, the mobility manager can send/receive information to/from network layer of protocol stack including the position information to control the nodes' movements for routing purpose. When the mobility manager decides to move, it generates a new move event and sends the event to the mobility controller. The mobility controller handles the move event and controls the movement of the node using the QualNet's API functions related to node mobility as shown in Figure 5.

### 5.2. Simulation Setup

In order to evaluate the performance of RoCoMAR, we consider a mobile sensor network where multiple regular nodes (e.g., sensors that are carried by unmanned vehicles or humans) are deployed. Those nodes sense environment to collect task-related data and send the data to the sink at a given position for further analysis. Since those nodes move cooperatively with other nodes to perform the duty (e.g., target tracking and search&rescue operation), they move in a group. In this paper, reference point group mobility model (RPGM) [38] is used to emulate movements of nodes, since RPGM can provide both the group mobility and a random mobility of each member in a group, which is well appropriate for tracking and monitoring operations in mobile sensor networks.

In RPGM, a group has a logical group center that moves according to group movement vector, which determines the group-wise movement. Each member node in a group is assigned with a reference point which determines the relative mobility of the node. Reference points move using the group movement vector. In addition, each member node has a random movement vector that allows the node to move randomly in the group. Note that movements of member nodes in the group are also limited by the group boundary. The random waypoint model (RWP) [6] is used to simulate both group-wise movements and member movements inside the group. In RWP, a node moves towards a randomly selected destination with a speed that is also randomly chosen in the range of minimum and maximum speeds. The movement is repeated after a randomly chosen pause time period.

The terrain dimension is 1000 meters × 1000 meters, and nodes are randomly placed in a circular group area with the radius of 400 meters. Network size in terms of the number of nodes is set to 25 for AODV, DSR, OLSR and GFR. On the other hand, for a fair comparison, the network contains 20 nodes and 5 RNs for RoCoMAR.

A network simulator QualNet 5.1, integrated with ConMosim, is used for simulation. In the scenario under consideration, mobile sensors transmit video data to the sink to share their findings and report the status. MPEG-4 is used for video data encoding, and in this paper, from 300 Kbps to 1.2 Mbps video streaming rate is used. In order to emulate the MPEG-4 data transmission, constant bit rate traffics with 1500 bytes data packets on top of UDP is used.

RoCoMAR is compared with AODV, DSR, OLSR and GFR routing protocols. IEEE 802.11 DCF is used as a MAC layer protocol. For the Physical layer, IEEE 802.11g radio is used with the maximum data rate of 54 Mbps. Also, the Rician model is used as a fading model to make the simulation environment more realistic. The average transmission radio range is set to approximately 225 meters. The average throughput and average end-to-end delay are chosen as performance metrics.

Some important simulation parameters are shown in the Table 1.

### 5.3. Simulation Results

In this subsection, we first present simulation results that show the end-to-end PDR gain in a simple network configuration. Then, in order to evaluate the network performance of RoCoMAR in a more realistic environment, we compare the performance of RoCoMAR with those of other routing protocols using the mobility model presented in Section 5.2.

#### 5.3.1. End-to-end PDR Gain in a Simple Network Configuration

In this subsection, we measure by simulation the end-to-end PDR gain when one relay node is placed at the mid-point position between the stationary source and destination nodes. Simulation setups presented in Section 5.2 are used except for network topology and node mobility. Both interference and non-interference cases are considered. The load of the source is set to 900 Kbps. For the case with interference, another source-destination pair, which has two intermediate nodes on the path, is placed in the neighborhood.

**Figure 6.**
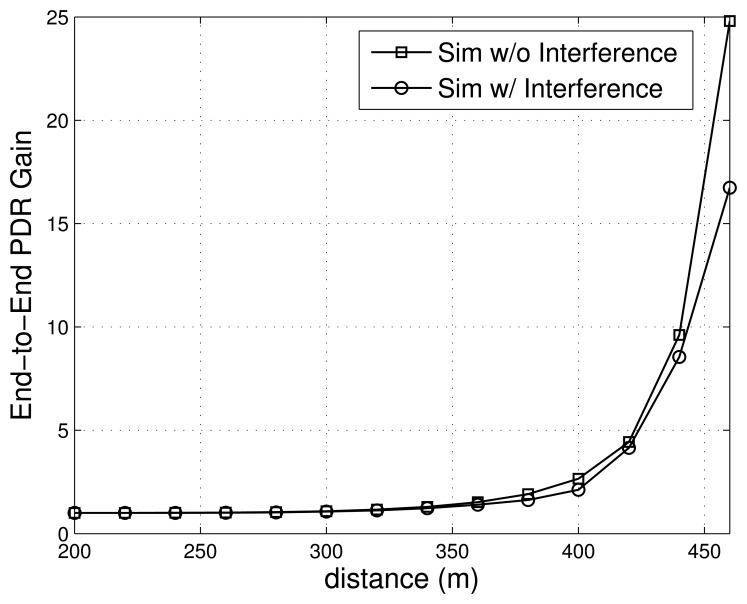
End-to-end PDR gain by using a robotic relay.

Figure 6 shows the end-to-end PDR gains as the distance between source and destination nodes varies. As shown in Figure 6, the gain increases as the distance grows. In particular, the gain sharply grows when the distance becomes longer then 350m. The results in Figure 6 also indicate that the gain without interference is higher than that with interference.

Note that the values of the gain in Figure 6 differ from those in Figure 4, since a different physical layer model is used. Although their absolute values are different, results from the simulations and the analytical model have a similar pattern, which fully verifies the motivation of our proposed algorithm.

#### 5.3.2. Simulation Results using a Realistic Scenario

In this subsection, we analyze the effects of node mobility, network load, and the number of data flows on the selected performance metrics: the average throughput and end-to-end delay. We also analyze the effect of transmission ranges and the parameter *ξ* on the performance of routing protocols.

##### (1) Node mobility

First, we analyze the effect of node mobility on the network performance. The movements of the group and each node inside the group is simulated according random waypoint model with the same pause time of 30 s. The group speed is randomly chosen in (*v_min_*,*v_max_*). The value of *v_min_* is set to 0 m/s (meter/seconds), and the value of *v_max_* varies from 10 to 20, 30, 40 m/s. The relative speed of nodes inside a group is randomly chosen in [0, 20] m/s. Also, RNs are assumed to have the constant speed of 20 m/s. Three data flows from source nodes to the sink are used for the network traffic; the load of each data flow is set to 900 Kbps.

**Figure 7.**
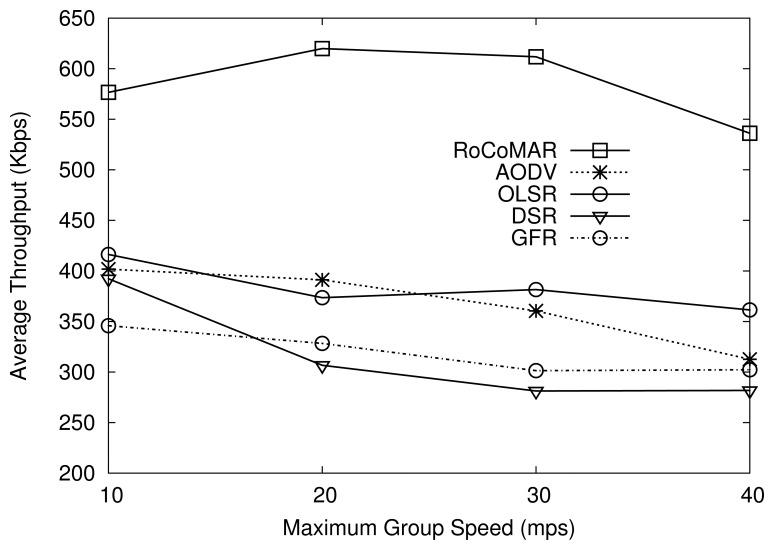
Effects of node mobility on average throughput.

As shown in Figure 7, RoCoMAR shows a higher throughput than AODV, OLSR, DSR and GFR. For example, when the maximum speed of the group is 20 m/s, RoCoMAR's throughput is higher than 600 Kbps, while other protocols show less than 400 Kbps. Also, over the variation of the maximum group speed, RoCoMAR consistently shows a higher throughput than other protocols. It is because RoCoMAR not only effectively reinforces the low quality links but also prevents wireless link breakage that causes a high loss rate of data packets and frequent route discovery processes. Also note that RoCoMAR enables RNs to keep the network connectivity between nodes in a group and the sink even when the group of nodes moves far away from the sink.

RoCoMAR's throughput improvement tends to decrease as the maximum group speed grows higher than 30 m/s as shown in Figure 7. One reason of this is that the limited speed of RNs. The RN with the speed of 20 m/s could not follow the mobile regular nodes if they are moving with the speed higher than the speed of RN. As also shown in Figure 7, in most cases, DSR shows the lowest throughput due to its stale route cache in high speed node mobility.

**Figure 8.**
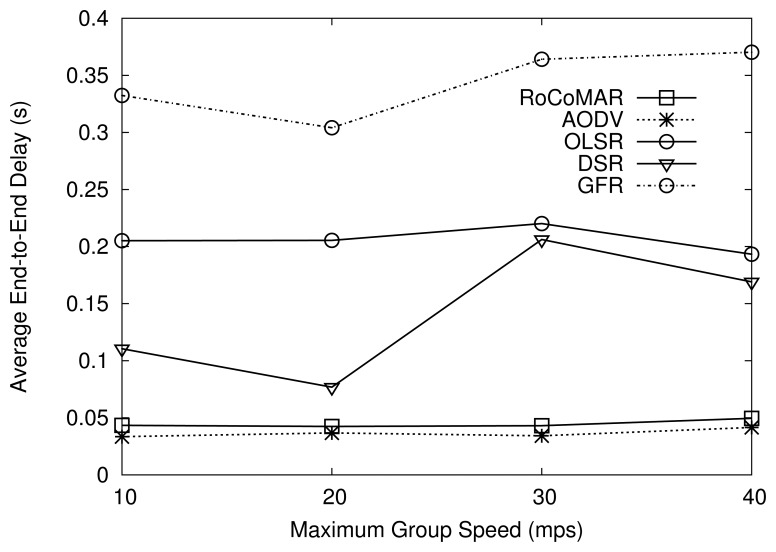
Effects of node mobility on average end-to-end delay.

Figure 8 shows the average end-to-end delay of routing protocols over variation of speed of the group. As shown in Figure 8, RoCoMAR has a lower delay than DSR, GFR, and OLSR, but a slightly higher delay than AODV. Note that the end-to-end delay of RoCoMAR is consistently shorter than 0.05 seconds, which is acceptable for most user applications (even for voice and video transmissions). Recall that RoCoMAR uses the same route discovery mechanism with AODV. Since relays can be added to the route, RoCoMAR can have a longer route in terms of the number of hops than AODV. This can result in a slightly high end-to-end delay compared with AODV.

GFR shows the highest average end-to-end delay compared with others, as shown in Figure 8. This is because neighboring nodes' position information can be stale, especially when nodes are moving at a high speed. The delay of DSR also highly depends on the group speed, as it needs to initiate a new time-consuming route discovery process when route cache becomes stale due to high node mobility.

##### (2) Network load

In order to be effective for a practical deployment, a network should be scalable in terms of network loads. To analyze the effect of the network load on the performance, RoCoMAR is compared with other protocols under various network loads. There are three data flows in the network, and each source transmits data at the rate from 300 Kbps to 1200 Kbps, which results in the total network load from 900 Kbps to 3.6 Mbps. The maximum speed of group and member movements are set to 20 m/s.

**Figure 9.**
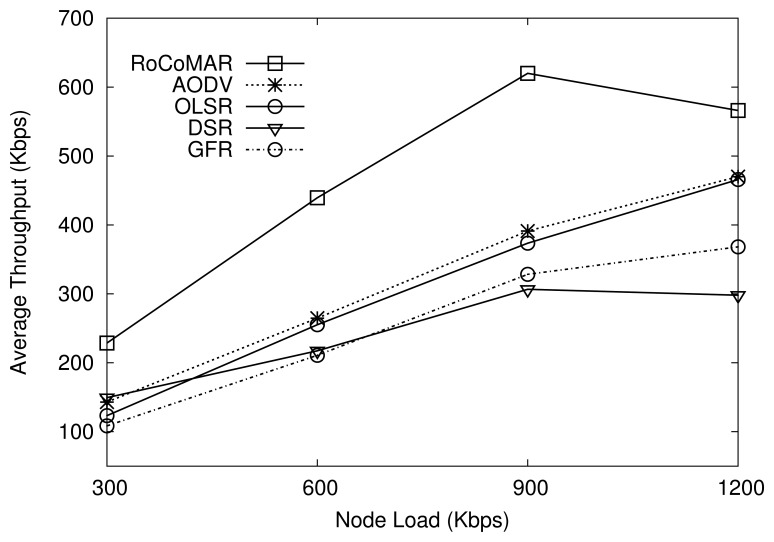
Effects of network traffic on average throughput.

As we can see in Figure 9, RoCoMAR achieves the highest throughput compared with others over various network loads. For example, when the load of each node is 900 Kbps, RoCoMAR has the average throughput over 600 Kbps, while other protocols show less than 400 Kbps. As also shown in Figure 9, RoCoMAR's throughput increases until the node load becomes 900Kbps then decreases. The reason is that a significant amount of MAC layer packet collisions and retransmissions occur as the data rate becomes close to the network capacity, which degrades the valid network throughput. DSR shows the lowest throughput when the network load is high.

**Figure 10.**
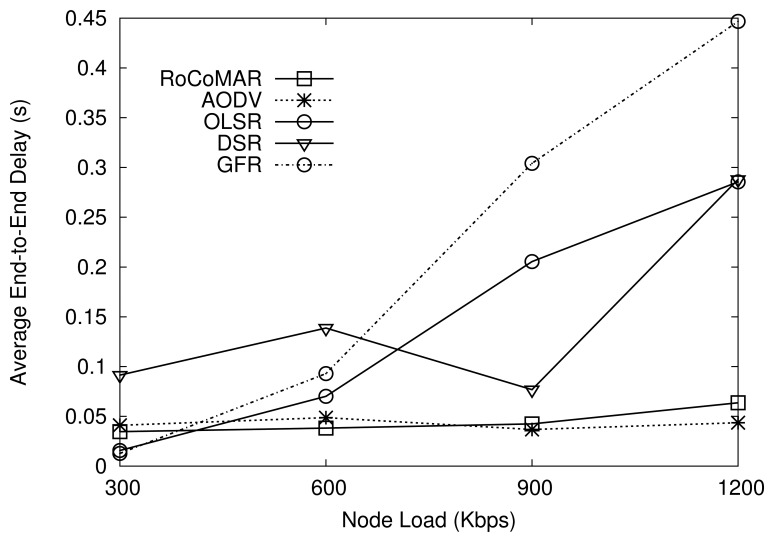
Effects of network traffic on average end-to-end delay.

Figure 10 compares the average end-to-end delay of RoCoMAR with other protocols. As shown in the Figure 10, RoCoMAR and AODV show a consistently low average end-to-end delay (less than 0.5 seconds) over the network loads. This indicates that RoCoMAR can be applied effectively without a large delay in a mobile sensor network where the high data rate applications are used. As also shown in Figure 10, the delay of GFR, DSR, and OLSR sharply increase as the network loads grow. In particular, the delay of DSR is fluctuating due to the unpredictable nature of DSR routing cache.

##### (3) Number of data flows

We also analyze the effect of the number of data flows on the network performance. The number of data flows is varied from 1 to 5 while keeping the node load as 900 Kbps. Recall that there are 5 RNs with the speed of 20 m/s. The maximum speeds of the group's movement and nodes inside the group are also set to 20 m/s.

**Figure 11.**
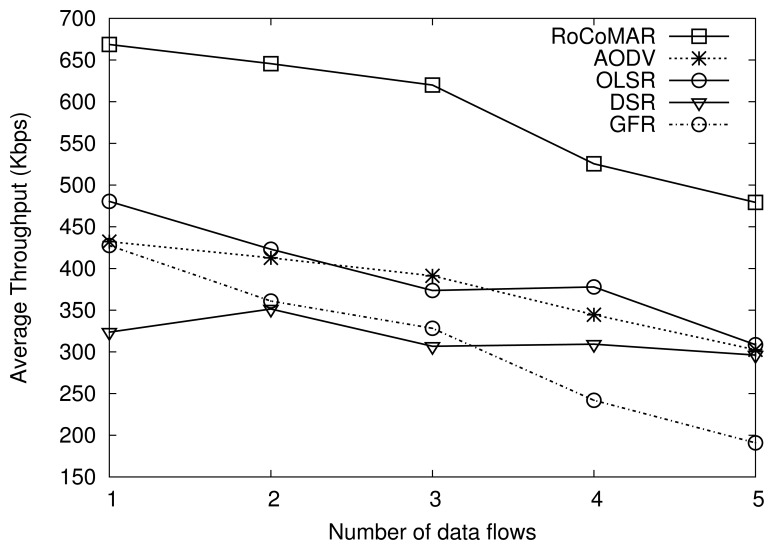
Effects of the number of data flows on average throughput.

As shown Figure 11, RoCoMAR also achieves a higher throughput than other routing protocols. For example, when the number of data flows is 2, RoCoMAR has throughput higher than 650 Kbps, while other protocols show less than 450 Kbps. As shown in Figure 11, the gap between throughput values of RoCoMAR and other protocols tend to decrease as the number of data flows grows. It is because there are only 5 RNs in the network, which implies that the ratio of the number of RNs per a route decreases from 5 to 2.5, 1.7, 1.25 and 1. As a result, it is possible that the poorest link is selected to be reinforced but the available RNs could not be found.

**Figure 12.**
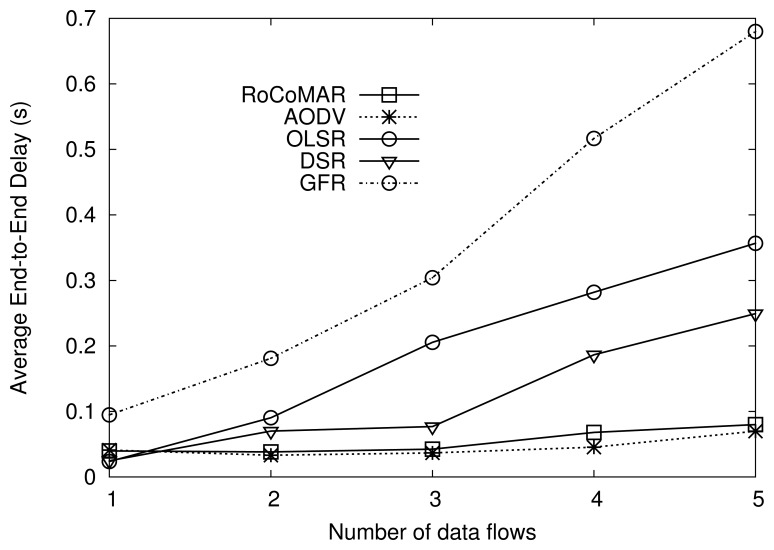
Effects of the number of data flows on average end-to-end delay.

Figure 12 shows the average end-to-end delay of protocols. As seen in Figure 12, RoCoMAR also shows the consistently low average end-to-end delay, while the delay of DSR, OLSR, and GFR becomes long as the number of data flow increases. The reason of low performance of those routing protocols is as following: the frequency of route discovery of those routing protocols is high because the quality of links varies unpredictably over time and space due to the fading channel and network topology changes. As a result, data delivery delay as well as the routing overhead are increased.

RoCoMAR alleviates the problem of low quality links and frequent route discovery. More specifically, when the quality of links on the route becomes poor, RoCoMAR detects this and places RNs until the quality of route meets the specific requirements, which makes the route more reliable and stable.

##### (4) Transmission ranges of nodes

In order to analyze the effect of the transmission range on the network performance, the radii of transmission range are varied from 200 m to 300 m. The maximum speed of group and member movements are set to 20 m/s. Each source transmits data at the rate of 900 Kbps. The terrain dimension is 1,000 m × 1,000 m and 25 nodes are randomly deployed in a circular group area with the radius of 400 m.

**Figure 13.**
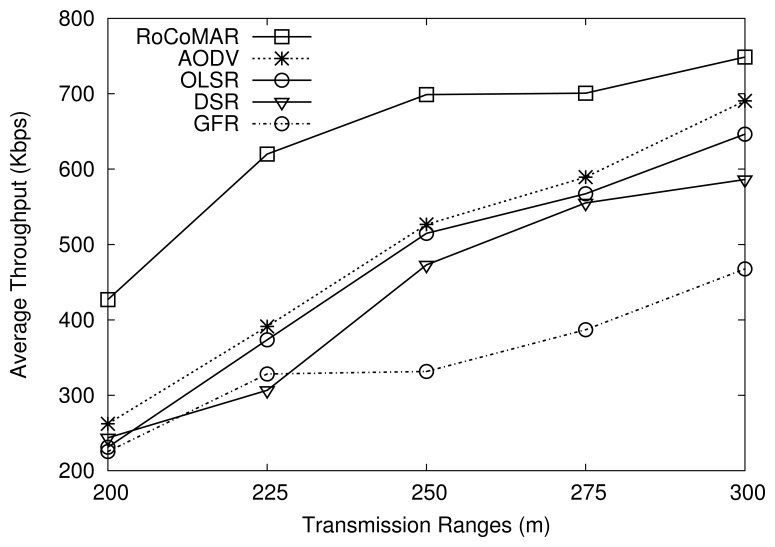
Effects of transmission range on average throughput.

Figure 13 shows the average throughput of routing protocols over different transmission ranges of nodes. As shown in Figure 13, the average throughput of all routing protocols increases as the transmission range of nodes grows. The reason is that, given the same distance between two communicating peers, a longer transmission distance leads to a good link quality due to a higher RSSI and SNR. Also, with a longer transmission distance, the possibility of link breakage becomes lower. The results in Figure 13 also indicate that RoCoMAR achieves the highest throughput among routing protocols over different transmission ranges. Moreover, RoCoMAR's throughput gain tends to decrease as the transmission range of nodes grows, since nodes can find a good quality link without link reinforcement i.e., the quality of links in the network becomes higher as the transmission range of nodes increases.

**Figure 14.**
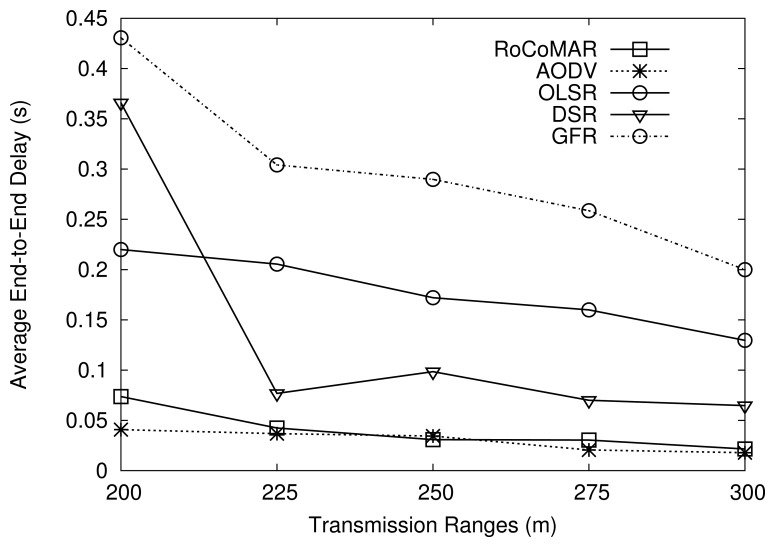
Effects of of transmission range on average end-to-end delay.

Figure 14 shows the average end-to-end delay of routing protocols over variation of transmission ranges. As shown in Figure 14, the end-to-end delay of routing protocols decreases as the transmission range of nodes becomes larger. It is because the routing protocol can establish a shorter route in terms of the number of hops when the transmission range is large. RoCoMAR and AODV show a low end-to-end delay compared with other routing protocols as shown in Figure 14.

##### (5) Effect of parameter *ξ* on the performance of RoCoMAR

As discussed in Section 4.1, the value of parameter *ξ* (xi) affects the performance of RoCoMAR. In order to understand the effects in detail, we collect the average throughput and average delay of RoCoMAR over different values of *ξ* in the simulation scenario where the load of each data flow is set to 900 Kbps. The maximum speed of group and member movements are also set to 20 m/s. The value of *ξ* varies from 0 to 0.6.

**Figure 15.**
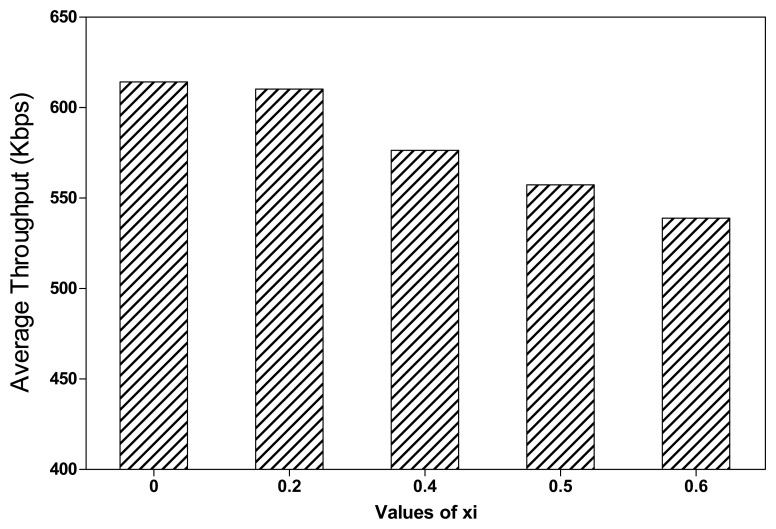
Effects of value of *ξ* on average throughput of RoCoMAR.

**Figure 16.**
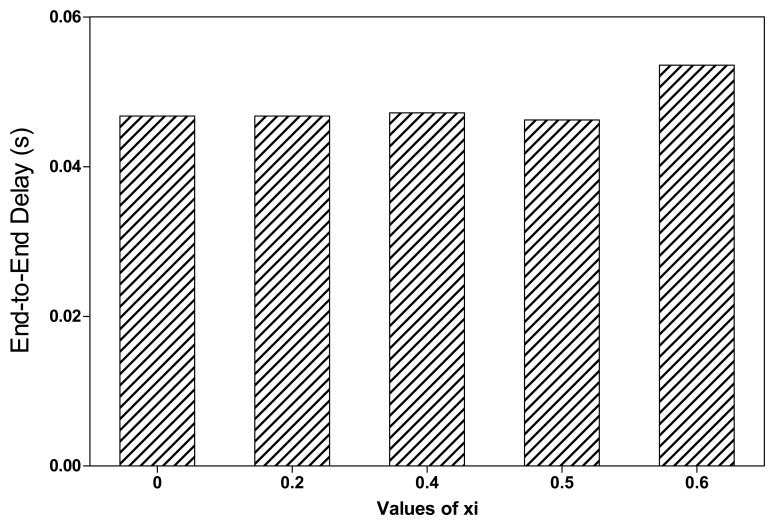
Effects of value of *ξ* on average end-to-end delay of RoCoMAR.

As shown in Figure 15, the average throughput of RoCoMAR decreases as the value of *ξ* grows. This is because a larger value of *ξ* results in a low possibility of placing a new relay node even some degree of performance gain is expected. Figure 16 shows the average end-to-end delay of RoCoMAR over different values of *ξ*. As shown in Figure 16, the delay of RoCoMAR mostly keeps stable as the value of *ξ* varies. The these results indicate the variation of *ξ* does not have a significant effect on the end-to-end delay of RoCoMAR.

## 6. Concluding Remarks

In this paper, we have proposed a novel ad hoc routing and relay architecture called RoCoMAR (Robots' Controllable Mobility Aided Routing) that uses robotic nodes' controllable mobility, in order to tackle the problem of low network performance and provide a desired end-to-end data transfer quality. RoCoMAR repeatedly performs link reinforcement process with the objective of maximizing the network throughput, where the link with lowest quality on the path is replaced with high quality links be placing a robotic node as a relay at an optimal position. Once placed as relay, the robotic node performs adaptive link maintenance by adjusting its position according to the movements of regular nodes. According to the simulation results, RoCoMAR outperforms existing ad hoc routing protocols for MSN in terms of network throughput and end-to-end delay.

For future work, we plan to design an analytical model to estimate the speed of link reinforcement considering RNs' distribution and moving speed. We also intend to extend RoCoMAR to find and establish an optimal path in terms of energy consumption and throughput in a network that consists of multiple RN and regular nodes. The recovery of failure of RNs will also be considered. In addition, we also plan to investigate optimal positions for RNs considering communication parameters such as RSSI and SNR.

## Figures and Tables

**Table 1.** Simulation Parameters and Values.

**Parameters**	**Values**
Simulation time	600 s
Mobility	RPGM model
Simulation area	1,000 m × 1,000 m
Group area radius	400 m
Transmission range	225 m
Traffic source	MPEG-4 Video Streaming (CBR/UDP)
Packet size	1,500 bytes
Routing protocols	RoCoMAR, AODV, OLSR, DSR, GFR
MAC layer	IEEE 802.11 DCF
Physical layer IEEE	802.11g
Maximum Bit rate	54 Mbps
Fading model	Ricean fading with K = 10
Number of nodes	25
Number of RNs	5
